# Finger motion and contact by a second finger influence the tactile perception of electrovibration

**DOI:** 10.1098/rsif.2020.0783

**Published:** 2021-03-31

**Authors:** Yasemin Vardar, Katherine J. Kuchenbecker

**Affiliations:** ^1^ Haptic Intelligence Department, Max Planck Institute for Intelligent Systems, Heisenbergstr. 3, 70569 Stuttgart, Germany; ^2^ Department of Cognitive Robotics, Faculty of Mechanical, Maritime and Materials Engineering (3mE), Delft University of Technology, Mekelweg 2, 2628 CD Delft, The Netherlands

**Keywords:** electrovibration/electroadhesion, touchscreen, haptics, perception

## Abstract

Electrovibration holds great potential for creating vivid and realistic haptic sensations on touchscreens. Ideally, a designer should be able to control what users feel independent of the number of fingers they use, the movements they make, and how hard they press. We sought to understand the perception and physics of such interactions by determining the smallest 125 Hz electrovibration voltage that 15 participants could reliably feel when performing four different touch interactions at two normal forces. The results proved for the first time that both finger motion and contact by a second finger significantly affect what the user feels. At a given voltage, a single moving finger experiences much larger fluctuating electrovibration forces than a single stationary finger, making electrovibration much easier to feel during interactions involving finger movement. Indeed, only about 30% of participants could detect the stimulus without motion. Part of this difference comes from the fact that relative motion greatly increases the electrical impedance between a finger and the screen, as shown via detailed measurements from one individual. By contrast, threshold-level electrovibration did not significantly affect the coefficient of kinetic friction in any conditions. These findings help lay the groundwork for delivering consistent haptic feedback via electrovibration.

## 1. Introduction

Researchers worldwide want to discover how to generate compelling tactile sensations on touchscreens to improve the usability of mobile devices, automotive control panels and many other interactive products. One technique for generating such sensations is using electrostatic actuation to control the interaction forces between the screen and the finger-pad of the user [1]. When an alternating voltage is applied to the conductive layer of a touchscreen, a periodic attractive force is generated between its surface and the user's finger (see electronic supplementary material, figure S1). Many people might have felt this salient effect when running their finger along a metal laptop case, lamp or other device connected to AC power. Systematic modulation of the alternating voltage creates various effects, giving rise to the haptic rendering approach commonly called electrovibration [1]. Although one needs to apply high input voltages (50–150 V peak) to generate a notable tactile sensation, the required current intensity is low [2]. Hence, electrovibration requires less power than methods that mechanically vibrate the screen to generate a tactile cue. Moreover, the electrostatic force occurs at the finger contact location, and hence it does not propagate vibration waves through the entire touchscreen or handheld device [1,3]. Overall, its fast, dynamic, high-bandwidth, highly scalable and noise-free performance [1] makes it a promising technology for future mobile phones, tablets, information displays and wearable devices. Nonetheless, even though generating a tactile sensation via this method is technically straightforward, the perceptual effects of electrovibration and the underlying physical mechanisms are not yet adequately understood.

The electrostatic attraction between human skin and a charged plate was discovered as a physical phenomenon by Johnson & Rahbek [4] and later separately by Mallinckrodt *et al*. [5]. About 20 years later, this effect was used by Strong & Troxel [6] to generate haptic feedback for the first time. They developed a tactile display consisting of an array of opaque electrodes insulated with a thin layer of dielectric, polyvinylidene chloride. They also proposed the first mathematical model based on the well-known parallel-plate capacitor theorem to describe the relationship between a given input voltage and the output electrostatic force pulling the finger toward the touchscreen. This model states that the resulting electrostatic force is proportional to the square of the voltage difference across the interface. Using a similar display, Kaczmarek *et al*. [7] found that humans are less sensitive to positive pulses of electrovibration than to negative or biphasic pulses; they explained that this disparity may be due to the asymmetric electrical properties of human skin.

Later Bau *et al*. [1] delivered electrovibration via a transparent commercial touchscreen, which demonstrated the potential of this technology for many modern applications. They also measured human sensory thresholds of electrovibration using sinusoidal inputs applied at different frequencies and showed that the threshold voltage follows a U-shaped curve as a function of frequency centred around 180 Hz. Meyer *et al*. [8] measured the contact forces caused by electrovibration and showed that they depend on input voltage frequency. By conducting psychophysical experiments and measuring contact forces, Vardar *et al*. [9] showed that low-frequency square-wave voltage signals are perceived more strongly than sinusoidal ones at the same frequency. They explained that even slow square waves have high-frequency components that stimulate the sensitive Pacinian psychophysical channel. In another study on electrovibration, Vardar *et al*. [10] showed that the perceived sharpness of virtual edges explored by sliding depends on the local haptic contrast between the background texture and the foreground item.

The cited studies on electrovibration perception consider almost only single-finger interactions where the user slides his or her dominant index finger across the screen. However, user interactions with current electronic devices include many different finger touch gestures, such as tapping, pressing, swiping and pinching. Hence, when electrovibration is used to provide tactile feedback on these devices, any sensation differences that depend on the performed touch interaction will be crucial for design specifications. As electrovibration is based on the electrical circuit formed by the touchscreen and the contacting skin, interacting with the surface using different motions [11] or multiple fingers [12,13] will affect the resulting circuit, as well as the forces and their perceived effects. For example, various sources [5,9,14] have anecdotally reported that electrovibration cannot be perceived when the finger is stationary, yet there is no systematic research to verify this statement or understand the underlying phenomenon. Similarly, it is still unknown how perceptual sensitivity changes due to multi-finger interactions. Thus, this paper aims to shed light on our limited knowledge of how finger motion (stationary or moving), finger normal force and contact by a second finger affect electrovibration perception.

For that purpose, we first conduct psychophysical experiments to measure 15 participants' absolute detection thresholds of an electrovibration stimulus along with the contact forces that occurred during their interactions. In the experiments, the participant explores the touchscreen using four different touch interactions: two fingers moving (2M), one finger stationary and another finger moving (1S1M), one finger moving (1M) and one finger stationary (1S), while applying two different normal forces (0.5 N and 1 N) per finger (see table 1 and electronic supplementary material, movie S1). These conditions represent the two main single-finger gestures (swiping and pressing) and their two pairwise combinations that involve movement. These experiments are complemented by measurements of the electrical impedance of the first author interacting with the touchscreen under the same experimental conditions to explain the potential physical mechanisms underlying the psychophysical results.

**Table 1.** Conditions for the psychophysical experiments.

touch interaction	non-dominant finger speed (mm s^−1^)	dominant finger speed (mm s^−1^)	normal force per finger (N)
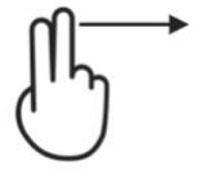 2M	—	50	1
	—	50	0.5
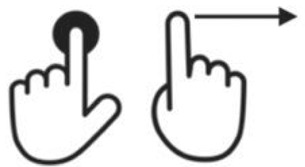 1S1M	0	50	1
	0	50	0.5
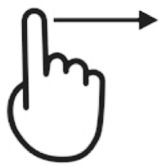 1M	—	50	1
	—	50	0.5
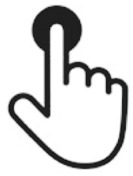 1S	—	0	1
	—	0	0.5

## 2. Methods

### 2.1. Psychophysical experiments

These experiments aimed to identify eight absolute detection thresholds, each of which is the minimum voltage that a participant can barely detect in a particular condition, along with the corresponding electrovibration forces. The subject's task in each trial was to contact the touchscreen with a specified touch interaction (2M, 1S1M, 1M or 1S) and normal force per finger (0.5 N or 1.0 N) over two successive intervals and then report whether they felt an electrovibration stimulus in the first or second interval.

#### 2.1.1. Participants

Eight women and seven men with an average age of 28.9 years (standard deviation, s.d.: 6.9) participated in the psychophysical experiments. None of them had current or past sensory–motor disabilities. One participant was left-handed, and three had previous experience with electrovibration technology. The experimental procedures were conducted in accordance with the Declaration of Helsinki and approved by the Ethics Council of the Max Planck Society under protocol no. 18-02A. All participants gave informed consent.

#### 2.1.2. Apparatus

**Figure 1.**
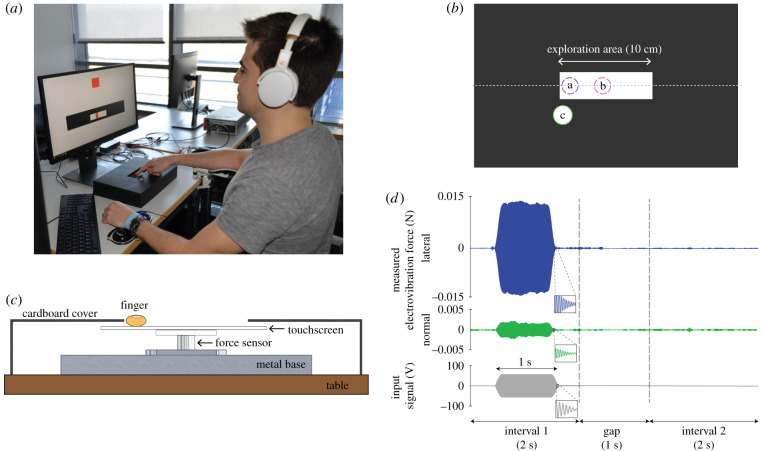
Apparatus and procedures of the psychophysical experiments. (*a*) A participant conducts an absolute threshold experiment using the one finger moving (1M) touch interaction. He synchronizes his motion with that of a visual cursor displayed on the LCD screen to make a single stroke from left to right across the touchscreen. The contact force and torque vectors are measured by a force sensor. (*b*) Illustration of the experimental apparatus from the top. The designated areas (a, b and c) indicate the initial finger locations for different touch interactions. The dotted grey line represents the cutaway line for the next diagram. (*c*) Cutaway illustration of the experimental apparatus from the front. (*d*) An example trial of the psychophysical experiment. The stimulus was generated by applying a 125 Hz sinusoidal voltage signal to the touchscreen during one of two temporal intervals, which were signalled to the participant on the screen. Each interval lasted 2 s, separated by a 1-s-long gap. The electrovibration stimulus was displayed in either the first or second interval randomly. In the interval without the electrovibration stimulus, the participant felt only the smooth glass surface. The participant gave his or her response after interval 2. In this example trial, the input voltage was applied during interval 1 and had a peak-to-peak amplitude of 100 V. The measured forces induced by electrovibration in the lateral and normal directions were calculated by band-pass filtering the contact forces between 240 and 260 Hz (double the sinusoid's frequency of 125 Hz).

During the experiments, the participant sat in front of a touchscreen and an LCD screen (figure 1*a*). The touchscreen (SCT3250, 3M Inc.) was attached on top of a force sensor (Nano 17 Titanium SI-16-0.1, ATI Inc., see figure 1*c*). The contact force and torque vectors were measured by this sensor and sampled by a data acquisition board (PCIe 6323, NI Inc.) at a rate of 10 kHz. The voltage signal applied to the touchscreen was first generated by a DAQ card (PCIe 6321, NI Inc.) and then augmented by an amplifier (9200A, Tabor Inc.) The participants wore an anti-static strap (PCS Inc.) on their stationary wrist for grounding, and they wore noise cancellation headphones to mask auditory cues. They were asked to synchronize their scan speeds with the motion of a visual cursor displayed on the LCD screen. Participants entered their responses through a numeric keypad.

#### 2.1.3. Stimulus

The stimulus was an oscillating electrostatic force generated by applying a sinusoidal voltage signal to the conductive layer of the touchscreen. Previous studies [2,4,9] showed that when a sinusoidal voltage signal without a DC offset is applied to a touchscreen, the resulting electrovibration force occurs at twice the input signal frequency. A sinusoidal voltage frequency of 125 Hz was chosen for this study to cause a force at 250 Hz (figure 1*d*), which is the frequency in the centre of the 200–300 Hz range where people are known to be highly sensitive to mechanical stimuli [15]. The input signal, a 125 Hz sinusoid with zero mean, was sent directly to the touchscreen without modulating its amplitude; the resulting stimulus was an oscillating electrostatic force at 250 Hz (figure 1*d*, enlarged inset views). The signal started and ended as ramps with 100 ms rise and fall times. This method enables smooth stimulation of the skin with the desired frequency. The duration of the stimulus was 1 s as measured between half-power points of the stimulus (figure 1*d*).

#### 2.1.4. Procedure

Each participant conducted experiments using the four different touch interactions shown in table 1: 2M, 1S1M, 1M and 1S. For each touch interaction two different finger normal force values were tested: 0.5 N and 1 N. Each participant completed the experiments in a different random order. Before each session, the participants washed their hands with soap and water and then dried them at room temperature. The touchscreen was also cleaned with alcohol before each session.

The stimuli were displayed in two temporal intervals, which were signalled to the participant using a graphical user interface (GUI) designed in Matlab. Each interval lasted for 2 s. The participant's task was to decide whether the electrovibration stimulus was in the first or second interval. The location of the stimulus was randomized in each trial. The amplitude of the input voltage signal was changed using the three-up/one-down double adaptive staircase method. This procedure estimates the threshold that has a 75% correct probability of detection [16]. One of the staircases (trials with odd numbers) started at an initial voltage with a high amplitude that was easily perceptible but not painful, whereas the other one (trials with even numbers) started at an initial voltage with an amplitude that was definitely not perceivable by the participant. If the participant gave three correct responses (not necessarily consecutively), the voltage level of the stimulus was changed by 5 dB to make the task more difficult. If the participant gave one incorrect response, the voltage level was changed by 5 dB to make the task easier. A change of the response from correct to incorrect or vice versa was counted as one reversal. After three reversals, the step size was decreased to 1 dB [10]. The experiment was stopped automatically when the reversal count reached four for each staircase. The threshold was calculated as the mean of the last six reversals (last three reversals of each staircase). The maximum peak amplitude of the applied voltage was set at 150 V to avoid dielectric breakdown of the air gap between the finger and the screen, which was previously observed around that voltage value [11] and which causes a different kind of tactile sensation. If the subject gave three consecutive incorrect responses at 150 V, the experiment was stopped automatically without recording the threshold level.

Approximately 35–60 trials were presented in each session until the threshold was reached. Before starting the trials, the participant received instructions and completed a training session. This training session enabled them to become familiar with the electrovibration stimulus and to adjust their finger scan speed and normal force with a visual cursor before the actual experiment. The participants were trained to apply a normal force within 25% of the desired value before starting each trial.

The experimental procedures varied based on the tested touch interaction:

— For the 1S condition, the participant was instructed to put his or her dominant index finger at the middle of the exploration area (b in figure 1*b*) when the start signal appears on the screen. Then, the participant was asked to keep the finger at the same location for 2 s. Afterwards, they were asked to raise their finger.
— For the 1M and 2M conditions, the participant was instructed to press their finger(s) at an initial point (a in figure 1*b*) when the start signal appears on the screen. Then, the participant was asked to move their finger(s) to the right in time with a moving cursor for 2 s. The speed of the cursor was 50 mm s^−1^. When one left-to-right stroke was finished, they were asked to raise their finger(s) and bring their hand back to the initial point.
— For the 1S1M condition, the participant was instructed to touch the index finger of their non-dominant hand at the designated place (c in figure 1*b*) and the other index finger at the initial point (a in figure 1*b*) when the start signal appears on the screen. The subjects were asked to move the index finger of their dominant hand to the right in time with a moving cursor for 2 s. The speed of the cursor was 50 mm s^−1^. When they finished one left-to-right stroke, they were asked to raise their dominant finger and bring their hand back to the initial point. The orientation of the cardboard cover was rotated 180° for the left-handed participant, who performed all strokes from right to left.

After experiencing the first interval, the participant repeated the same procedure. They then reported whether they felt the stimulus in the first or second interval.

Each participant completed the experiments in eight sessions (4 conditions × 2 normal force levels), which could be executed on separate days. The duration of each session was about 15–20 min.

#### 2.1.5. Data analysis

The absolute threshold voltages indicate the input voltage required to generate the minimum perceivable electrovibration force for that subject in that condition. Thus, the voltages applied in the six threshold trials were averaged to obtain the relevant threshold voltage.

The collected force signals from the same sets of six trials at the threshold level were then analysed. The average normal component of the unfiltered forces for each participant within each experimental condition was calculated to document the normal forces that were actually applied. The individual contributions of the stationary and moving fingers for the 1S1M condition were calculated by assuming each finger made point contact with the touchscreen (see electronic supplementary material, text A for derivation). Previous studies [17,18] used this assumption to estimate the contact location of a finger or tool on the surfaces of an object rigidly attached to a force/torque sensor.

The actual stimulus that induces the perception is the electrovibration force at the threshold level. As a 125 Hz sinusoidal voltage was applied to the conductive layer of the touchscreen, the electrovibration force occurred at 250 Hz. The amplitude of this oscillatory force was calculated by first manually segmenting the force signals to keep the contact forces only when electrovibration was present. Then, the segmented signals were band-pass filtered between 240 and 260 Hz. After that, the average power of each filtered signal was calculated and multiplied by two. The square root of this value gave the amplitude of the electrovibration force.

To test the reliability of our measurements, we calculated the average force signal energies at 250 Hz at the threshold level with and without electrovibration. The energy per unit time of the electrovibration force, *E*_250Hz_, was first calculated by integrating its power spectrum. The same procedure was applied for the intervals where electrovibration was absent, yielding *N*_250Hz_. Then, the log of their ratio was calculated for each trial using the formula log_10_ (*E*_250Hz_/*N*_250Hz_) in both the lateral and the normal directions. This ratio is zero when two energy values are the same.

Similarly, the collected force signals from all trials (not just the six trials at threshold) were analysed to enable calculation of the electrovibration force amplitudes across input voltages. For this analysis, the powers of the force signals that occurred in the lateral and normal directions were summed. The peak magnitude of the electrovibration force was found by multiplying the average power of this signal by two and then taking the square root. Then, a mathematical function ($F_{250\mathrm{Hz}}=kV_{125\mathrm{Hz}}^{2}$, where *F*_250Hz_ is the electrovibration force, *V*_125Hz_ is the applied voltage and *k* is a constant) was fit to the results of each experimental condition.

The average kinetic friction coefficient during the application of electrovibration was calculated from the intervals that belong to the trials at the threshold level for the three conditions involving finger motion. For each of these trials, the force recording was first segmented such that it has two parts: with and without electrovibration. Then, for the part of the trial with electrovibration, the unfiltered lateral force segment was divided by the normal force segment and averaged. The average friction coefficient for each participant for each condition was calculated by averaging the coefficients obtained from the six trials at the threshold level. The friction coefficients without the electrovibration force were calculated by applying the same procedure to the parts of the trials without electrovibration.

### 2.2. Electrical impedance measurements

These measurements were conducted to understand the changes in the electrical circuit formed between the user's finger(s) and the touchscreen due to the applied touch interaction and normal force. One right-handed participant (first author) conducted these measurements. The apparatus was similar to the one used in the psychophysical experiments. However, the input voltage was generated by an impedance analyser (MFIA, Zurich Instruments) and sent to the touchscreen without amplification due to the voltage limitation of the impedance analyser. The grounding electrode was connected to the participant's left wrist. The measurements were conducted using two terminals of the impedance analyser. Before the experiments, the system was calibrated (see electronic supplementary material, text B for details).

During the experiments, the participant was trained to keep her finger velocity constant at 50 mm s^−1^. She also adjusted her finger force with a visual cursor similar to the one in the perceptual experiments. The impedance of the touchscreen–finger circuit was measured for the same eight conditions (4 touch interactions × 2 normal forces) tested in the psychophysical experiments. Each measurement was repeated five times.

## 3. Results

### 3.1. Psychophysical experiments

**Figure 2.**
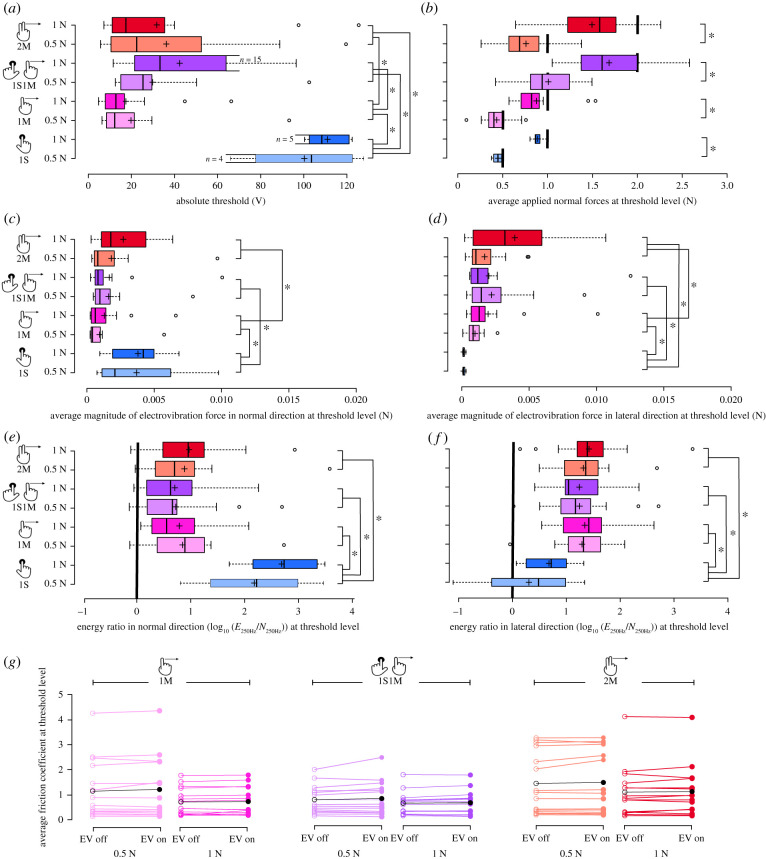
Results of the psychophysical experiments. Box plots of the (*a*) average threshold voltages, (*b*) average applied normal forces at threshold, (*c*) average total magnitude of the electrovibration forces in the normal direction and (*d*) in the lateral direction at threshold, (*e*) energy ratio of the 250 Hz signal with and without electrovibration in the normal direction and (*f*) in the lateral direction. The results corresponding to each experimental condition are colour-coded. The centre lines show the medians; box limits indicate the 25th and 75th percentiles. The whiskers extend to 1.5 times the interquartile range. Outliers are represented by circles (o), and plus signs (+) represent sample means. The thickness of each bar is proportional to its sample size (*n*), as indicated on (*a*). The connected brackets with stars (*) mark statistically significant pairs. (*g*) Average kinetic friction coefficients at threshold level with and without electrovibration (EV) for each subject. The average friction coefficients across subjects are indicated in black for each experimental condition.

The measured absolute detection threshold voltages of the participants are shown in figure 2*a*. Only four of the 15 participants could feel the electrovibration stimulus at the highest tested voltage levels for the 1S case with low force; five felt it at high force. A generalized linear mixed model (GLMM) was created to test the effects of applied touch interaction and force on the detection thresholds [19]. The voltage thresholds were significantly affected by the applied touch interaction (*p* < 0.001) but not by the applied normal force. A sequential Bonferroni corrected *post hoc* test indicated that the means of the voltages were significantly different (*p* < 0.05) for all interaction pairs except 1S1M and 2M.

The average normal forces that participants applied during the intervals at the threshold level are depicted in figure 2*b*. Paired *t*-tests showed that the participants applied significantly different normal forces for low (0.5 N) and high (1 N) force conditions (*p* < 0.001). However, the mean values were less than the desired levels for each condition except 1S1M at low force (compare the mean values (+) with thick black lines depicted in figure 2*b*). The contributions of the two different fingers for the 1S1M condition were also analysed. The average contributions of the moving and stationary fingers were calculated as 0.55 N and 0.45 N for the low-force condition and 0.80 N and 0.87 N for the high-force condition, respectively. Bonferroni corrected *t*-tests showed that the contributions of the sliding and stationary fingers to the total applied force were similar in both low- and high-force conditions.

**Figure 3.**
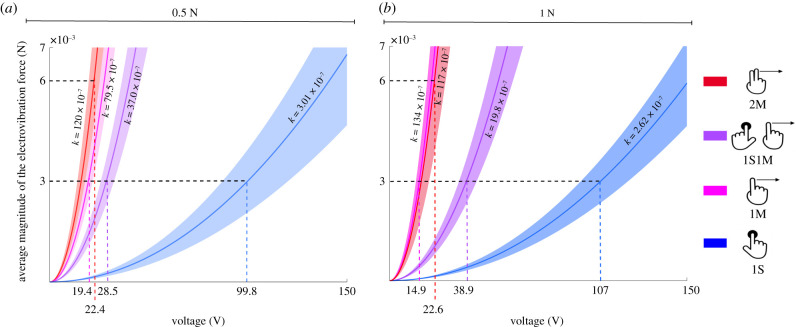
The average electrovibration force magnitudes measured during the psychophysical experiments. The functions, $F_{250\mathrm{Hz}}=kV_{125\mathrm{Hz}}^{2}$, fit to average peak magnitude of total electrovibration force, *F*_250Hz_, versus the input voltage, *V*_125Hz_, for (*a*) low and (*b*) high normal force conditions. The results corresponding to each experimental condition are colour-coded. The bold lines and shaded regions indicate the mean and standard error (s.e.m.) of fit functions across subjects, respectively. The average fit coefficients, *k*, are indicated on the graph of each experimental condition. The black dashed line indicates an arbitrary electrovibration force value (3 mN) that can be generated in all conditions where perception occurs due to one finger. We doubled this value to represent the same force for the 2M case. The input voltage values needed to obtain that force value in each experimental condition are shown along the *x*-axis.

Figure 2*c*,*d* depicts the average magnitude of the electrovibration forces calculated at the threshold level. Analysing these results using GLMM showed that the total electrovibration forces in the normal and lateral directions were affected by the applied touch interaction but not by the applied normal force (*p* < 0.05). A Bonferroni corrected *post hoc* test revealed that the electrovibration forces in the lateral direction were significantly lower for the 1S condition compared to the other conditions. Also, both lateral and normal forces measured for the 2M condition were significantly higher than the same for the 1M condition (*p* < 0.05). A GLMM analysis was also conducted on the coefficient *k* of the functions fitted to the average magnitude of the electrovibration forces measured from all trials (figure 3). The coefficients were affected by the applied touch interaction but not by the applied normal force (*p* < 0.05). A least significance difference *post hoc* test showed that all the coefficients were different from each other except the ones for the 1M and 2M conditions.

Electrovibration generated significant differences in all calculated energies for both normal and lateral directions (compare the mean values (+) with the thick black lines in figure 2*e*,*f*). GLMM analysis showed that these ratios were affected by applied touch interaction but not by applied normal force. A Bonferroni corrected *post hoc* test revealed that the energy ratios in the normal direction for the 1S condition were significantly higher than the same values for other conditions. Simultaneously, the 1S values in the lateral direction were significantly lower than the other conditions.

GLMM analyses were conducted on the average kinetic friction coefficients at threshold level for both the presence and absence of electrovibration (figure 2*g*). The friction coefficients were affected significantly by the applied touch interactions, but not by the applied force, regardless of the presence of electrovibration. The effect of electrovibration on the resulting kinetic friction coefficients was also evaluated by a paired *t*-test. Electrovibration increased the average friction coefficient between the finger and the touchscreen by 2.72%, but this increase was not statistically significant (compare filled and empty circles in figure 2*g*).

### 3.2. Electrical impedance measurements

**Figure 4.**
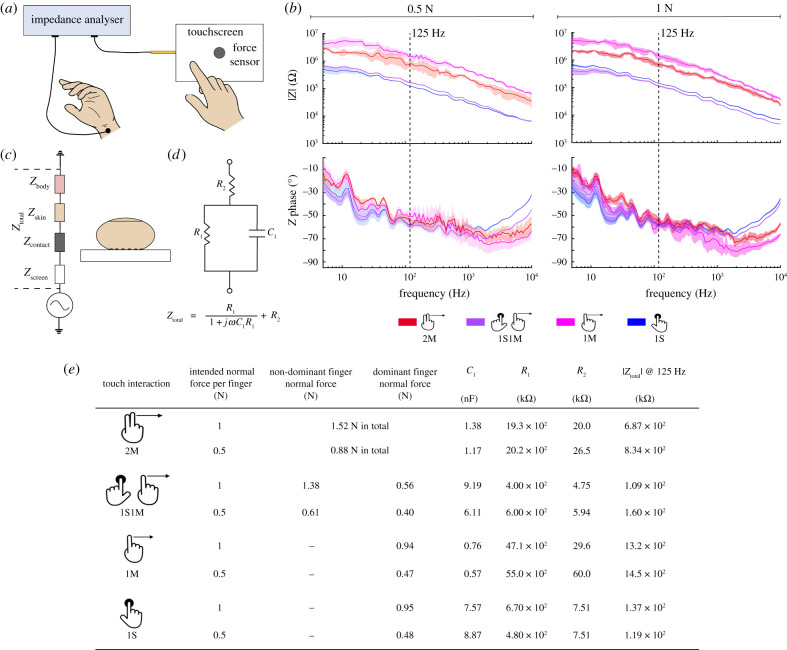
Apparatus and results of the impedance measurements. (*a*) Illustration of the experimental set-up used for impedance measurements. (*b*) The results of the impedance measurements, colour-coded by experimental condition. The shaded regions correspond to standard errors (s.e.m.) of five measurements for each condition. (*c*) The lumped electrical model of the finger–touchscreen system. (*d*) The total impedance can be modelled as one resistance connected in series with another resistance in parallel with a capacitance. (*e*) Electrical model fit to the impedance results, measured average normal forces during the measurements and absolute overall impedance values measured at 125 Hz.

The results of the electrical impedance measurements are shown in figure 4*b*. The total impedance of the touchscreen–finger system, *Z*_total_, can be represented as the impedance of the user's body, the finger skin, the touchscreen and the contact between those two (figure 4*c*) connected in series [11]. The system's electrical behaviour was elucidated by fitting models to the measured impedance values [20,21]. *Z*_total_ can be modelled as a resistance, *R*_1_, in parallel with a capacitance, *C*_1_, with this pair connected in series to another resistance, *R*_2_. Figure 4*e* shows the resulting model parameters as well as the forces exerted during the measurements.

## 4. Discussion

### 4.1. Importance of lateral motion

The results show that the input voltage required to create an arbitrary electrovibration force value (peak force magnitude at 250 Hz) is significantly lower when one finger moves across the screen than when it is stationary (compare the 1M and 1S fit coefficients, *k*, in figure 3). Accordingly, most subjects could not feel the electrovibration stimulus at any tested voltage value for the 1S condition; however, in contradiction to prior anecdotal reports [5,9,14], about 30% of the subjects could reliably detect electrovibration without finger motion. As would be expected from the diminished force production, the absolute threshold voltages of these subjects were significantly higher in 1S than for all touch interactions with finger motion (figure 2*a*).

The augmentation of the forces can be explained by the additional shear forces that occurred during motion due to the finger's elastic behaviour [22–24]. When an alternating input voltage is applied to a touchscreen, the resulting attraction forces will result in brief sticking intervals between the fingertip and the touchscreen. For the stationary finger, the measured forces will represent the strains in the normal direction caused solely by this attraction force. However, for the sliding finger, bulk lateral displacement will create additional shear strains. When the attraction force decreases near the zero voltage instants and then disappears, the shear strains pull the finger back, causing larger changes in the reaction forces in both the lateral and normal directions. This phenomenon will cause even larger measured forces when the finger slides faster, as observed in previous studies [9,25].

Another factor for the higher force and energy thresholds for the stationary finger compared to the sliding finger could be the dependence of human psychophysical sensitivity on the stimulus direction. Brisben *et al*. [26] showed that the detection thresholds of human participants for high-frequency vibrations were 30% lower when the stimulus direction was parallel to the skin (as when sliding along a surface with electrovibration) than when it was perpendicular (as when holding the finger stationary on an electrovibration stimulus). They explained that tangential skin vibration may produce shear strain in the subcutaneous tissues more effectively than perpendicular skin vibration [27].

In addition to the measured forces, the measured electrical impedance values are also higher for the cases with sliding motion (figure 4). Impressively, the measured electrical impedance for the 1S case is almost 10 times lower than the 1M case. Similar results were obtained by Shultz *et al*. [11]. They hypothesized that this impedance drop in the stationary condition occurs because the air gap at the contact between the finger and the touchscreen fills with sweat, which is both highly conductive and has a higher dielectric constant than air, dramatically lowering the contact impedance. The lower contact impedance decreases the finger's accrued effective voltage and resulting electrostatic force, making the stimulus difficult to feel. We further hypothesize that the finger relaxations due to large strain variations during movement may reduce the finger's real contact area, therefore, also increasing the air gap.

### 4.2. Two-finger interactions

The absolute voltage thresholds of the participants were significantly higher for the two-finger touch interactions than for the single-finger moving case (figure 2*a*). This finding can be explained by the electrical changes in the finger–touchscreen circuit. As the touchscreen consists of only one large electrode and one insulator, the resulting electrovibration stimulus is not localized: all interacting fingers are exposed to the same voltage input. The corresponding electrovibration stimulus for each finger is bound to its accrued effective voltage. Hence, when two fingers interact with the touchscreen, the impedance of the finger is added in parallel to that part of the initial circuit, decreasing the overall impedance of the fingertip–touchscreen system [12,13]. In this study, the overall impedance measured for the 1M case is almost twice the impedance for the 2M case, and 10 times the impedance for the 1S1M case (compare pink, red and purple lines in figure 4*b*).

The parameters of the electrical models give more insight into the changes in the electrical characteristics of the finger–touchscreen system. Compared to the 1M case, the capacitance, *C*_1_, increased, and the resistances, *R*_1_ and *R*_2_, decreased for the 2M and 1S1M cases (figure 4*e*). This behaviour can be explained by the increase in the skin–touchscreen contact area with the second finger. Although the same behaviour was observed for the 1S1M case compared to the 1S case for the high-force condition, the trend was different when the applied forces were lower. That phenomenon might be caused by the differences in the applied normal forces between the 1S1M and 1S cases (compare the normal forces in figure 4*e*, rows 4 and 8) or sweating [28].

Although the absolute voltage thresholds were higher for the two-finger conditions, the resulting force thresholds showed a different trend (figure 2*c,d*). There was not a significant difference between the force thresholds of the 1S1M and 1M cases. Yet, those for 2M were almost twice the others. These results can be interpreted to mean that when there is lateral finger movement, participants needed a similar amount of electrovibration force for each moving finger for sensory detection. Such a phenomenon has also been observed in previous studies conducted with a variety of other haptic devices [29,30].

Moreover, the presence of the stationary finger for the 1S1M case did not affect the force thresholds of the participants when they explored the surface using only their index finger (compare purple and pink bars in figure 2*c*,*d*). As the voltage thresholds of stationary fingers were far higher than those for sliding (compare blue and pink bars in figure 2*a*), it can be assumed that subjects perceive the electrovibration force only under their moving finger.

### 4.3. Effect of applied normal force

The measured voltages and their corresponding force thresholds were not significantly affected by the applied normal force. This perceptual invariance might be caused by the fact that 0.5 N and 1 N are considered to be light and moderate touch, respectively, [31] and thus had similar finger mechanics. Interestingly, there is no consensus about the effect of applied normal force on electrovibration perception in the literature. Some studies reported that applying higher normal forces enhances electrovibration perception [32], but other studies reported the opposite trend [25,33]. Hence, further investigation is required to understand the effect of normal force on electrovibration perception.

Applying higher normal forces caused a slight decrease in the total impedance values for the cases that involve lateral motion. The system becomes less resistive and more capacitive (check *R*_1_, *R*_2_ and *C*_1_ in figure 4*e*). This behaviour may be caused by the increase in the finger contact area due to the higher normal force. However, for the single-finger stationary case, this trend disappears; applying higher normal forces increased the total impedance. Several factors such as sweating [14,34], changes in the contact area [14,35] and the thickness of the stratum corneum layer [36] could cause this change.

### 4.4. Detection of electrovibration stimulus

The results showed no significant changes to friction coefficients with and without electrovibration at the threshold level (figure 2*g*). Yet, the electrovibration generated significant differences in calculated force energies at 250 Hz (figure 2*e*,*f*). This finding shows that the main factor underlying the detection of electrovibration is not the increase in the magnitude of the friction but rather its high-frequency components [9]. This result might seem to contradict previous studies [8,37] that reported a notable increase in friction coefficients in the presence of electrovibration when high voltage values (greater than 140 V) were applied. There is no contradiction because the friction coefficients reported in this manuscript were calculated at the threshold level, where the average input voltage was low (less than 60 V).

### 4.5. Variability

Although the experimental conditions were constant for all participants, we observed large variations in detection thresholds, measured contact forces and friction coefficients. These variations are most likely caused by variations in the electrical and mechanical properties of human skin, human psychophysical sensitivity, skin moisture, skin temperature and finger size [23,38–40].

### 4.6. Implications for future electrovibration displays

Due to the changes in the electrical circuit of the finger–touchscreen interaction, maintaining stable electrovibration perception will be challenging for future electrovibration displays. A scenario where a user enjoys vivid tactile sensations can suddenly turn into frustration when a second finger touches the screen. One solution to this problem could be detecting the applied touch interaction by measuring the electrical impedance of the system [12]. This way, the voltage input can be adjusted based on the desired current level. Current-controlled systems can also prevent variability due to clothing and grounding [11,41]. Another approach for eliminating variability due to clothing, moisture or grounding can be rendering desired sensations via a closed-loop system by using the measured contact forces as feedback [42]. However, this technique cannot overcome the changes due to applied touch interactions unless it also detects them simultaneously.

This research used a commercial display as a touchscreen. This commercial display is produced for sensing purposes without considering tactile rendering applications. In the future, new touchscreens could be designed to maximize the force output with less voltage or current input by optimizing the insulator and surface roughness parameters [35]. As some participants could perceive electrovibration at high input voltages in the 1S condition, the optimization of the screen may be a promising way to convey effects when the finger is stationary.

### 4.7. Limitations and future extensions

The contact forces reported in this paper were measured by a force sensor placed under the touchscreen. If an electrostatic attraction force had occurred between the touchscreen and a non-deformable object resting on its surface, the net force transmitted to the force sensor would be zero. However, as the human finger is deformable and supported by the arm, the alternating electrostatic force causes deformations of the fingertip. Hence, forces caused by these deformations are transmitted to the sensor below. Nonetheless, the best way to evaluate the physical effects of electrovibration would be high-resolution imaging of finger–touchscreen contact [25,43].

Even though our experimental results showed that finger motion significantly increases the finger deformations induced by the electrostatic force, which decreases the corresponding detection thresholds, further measurements are needed to clearly explain the underlying physical phenomenon. More experiments should also be performed with a stationary finger at higher voltages to elucidate the factors that determine the detectability of electrovibration in this condition.

Finger moisture and contact area were not measured during the psychophysical experiments and impedance measurements. It is well known that moisture can affect finger mechanical properties [24] and the resulting electrostatic force [11,14,43]. One interesting approach could be measuring the moisture level of the finger over time during stationary and sliding conditions using a sensor that is integrated into the touchscreen.

Although the individual contributions of the stationary and moving fingers to the total normal force were calculated for the 1S1M case, this calculation is based on the assumption that both fingers are in point contact with the touchscreen [17,18]. The stationary finger was also assumed not to undergo any lateral movement. Moreover, the individual contributions of each finger for the 2M case could not be measured; they were assumed equal. Finally, this paper reports only the mean values of the participants' applied forces and tests their effect on the threshold values. The normal force variations during trials were not analysed, and they might have influenced the results. The possible consequences of these variations could be investigated in future work.

## Data accessibility

The data and code for the key figures in the manuscript can be found in the Dryad repository with the identifier https://doi.org/10.5061/dryad.9ghx3ffgp [44].
